# Short-term efficacy evaluation of artificial intelligence-based three-dimensional reconstruction of chest CT in segmentectomy: a propensity score-matched study

**DOI:** 10.3389/fonc.2025.1712040

**Published:** 2025-12-10

**Authors:** Xindong Luo, Ziqiang Wang, Wanrong Kang, Yunjiu Gou, Honglai Zhang

**Affiliations:** 1 The First Clinical Medical College, Gansu University of Chinese Medicine, Lanzhou, China; 2 Department of Thoracic Surgery, Gansu Provincial Hospital, Lanzhou, China; 3 Department of Anorectal, Gansu Provincial People’s Hospital, Lanzhou, China; 4 Gansu Provincial People’s Hospital, Gansu Provincial Clinical Medical Research Center for Anorectal Diseases, Lanzhou, China

**Keywords:** artificial intelligence, 3D reconstruction, hook-wire, pulmonary function, retrospective analysis, lung cancer

## Abstract

**Introduction:**

Artificial intelligence (AI)-based 3D reconstruction software is increasingly used in clinical practice. This study compared its clinical value with CT-guided Hook-wire localization in segmentectomy.

**Methods:**

A retrospective analysis was performed on 257 patients who underwent video-assisted thoracoscopic segmentectomy at Gansu Provincial People’s Hospital from January 2023 to August 2024. Patients were divided into an AI-3D group (134 cases, receiving preoperative AI-assisted 3D reconstruction) and a Hook-wire group (123 cases, undergoing Hook-wire localization).

**Results:**

Propensity score matching ensured comparability, with 97 pairs matched. Perioperative indicators, pulmonary function, and quality of life were evaluated. After matching, the Hook-wire group had 2 pneumothorax and 4 hemothorax cases, while the AI-3D group had none. The AI-3D group showed significantly shorter operative time, chest tube indwelling time, and hospital stay, along with less intraoperative blood loss and drainage volume. No significant differences were found in postoperative complications or lymph nodes dissected. Six months post-surgery, the AI-3D group had better pulmonary function and quality of life scores.

**Conclusion:**

Thus, AI-based 3D reconstruction enables safe, effective segmentectomy with minimal impact on pulmonary function, showing good clinical value.

## Introduction

Lung cancer is the leading cause of cancer-related deaths worldwide (1). With the widespread adoption of low-dose chest computed tomography (CT) in lung cancer screening, the detection rate of early-stage lung cancer manifested as pulmonary nodules has been increasing annually. In recent years, the value of video-assisted thoracoscopic surgery (VATS) segmentectomy for such lesions has undergone re-evaluation. For peripheral lung cancers with a diameter of ≤2 cm on CT, segmentectomy has demonstrated comparable efficacy to lobectomy (2). Moreover, it allows for greater preservation of healthy lung tissue (3). Compared with lobectomy, pulmonary wedge resection, and other surgical procedures, segmentectomy requires the identification and dissection of more delicate bronchi and their accompanying vessels, followed by selective ligation. Given the complex and variable anatomy of the pulmonary segments, with numerous anatomical variations (4, 5), incorrect ligation may lead to massive hemorrhage, necessitating conversion to thoracotomy. Therefore, the rapid and accurate identification of pulmonary nodules becomes crucial. In clinical practice, CT-guided Hook-wire localization of pulmonary nodules is commonly employed; however, as an invasive procedure, it can result in complications such as pneumothorax and hemothorax (6). Consequently, the development of a safer, more accurate, rapid, and non-invasive method for localizing pulmonary nodules represents an urgent clinical challenge.

Anthropomorphic three-dimensional (3D) models are frequently utilized in clinical practice as adjuncts for preoperative planning and intraoperative navigation. Interactive 3D models have been demonstrated to significantly enhance surgeons’ stereoscopic perception and reduce surgical complications by enabling preoperative prediction of anatomical structures (7, 8). Previous studies have found that 3D models can accurately predict the resection volume and margins in liver surgery (9) and substantially reduce the abnormal rate of femoral anteversion restoration in hip replacement surgery (10). Nakashima et al. (11) were the first to report the use of 3D pulmonary reconstruction for planning and completing segmentectomy (LS6), observing a variant A6c in both the preoperative 3D reconstruction images and during the surgery.

Three-dimensional reconstruction models of chest CT can be created using semi-automatic tools (such as MIMICS^®^). These tools can simulate anatomical structures, clearly delineate pulmonary segment divisions, and determine the location of lesions. Traditional semi-automatic 3D reconstruction systems rely on manual threshold adjustments by physicians, achieving a segmentation accuracy of only 68% for complex anatomical structures (such as nodules at the edges of pulmonary segments) or subsolid nodules. The requirements for professional expertise, manual annotation, and high time consumption may dampen enthusiasm for the widespread adoption of these tools. Additionally, the inevitable introduction of subjective bias due to manual organ selection can lead to deviations in research outcomes (12–14). In contrast, the multi-resolution Volume Bottleneck Network (VB-Net) architecture-based chest CT 3D reconstruction system employed in this study has, to a certain extent, addressed the limitations of current manual 3D reconstruction methods. This system utilizes an end-to-end training framework, integrating multi-resolution strategies, optimizing loss functions, and refining preprocessing and postprocessing workflows to support fully automated and precise segmentation of organs in medical imaging.

This study focuses on thoracoscopic segmentectomy, comparing the clinical application effects of AI-assisted 3D reconstruction for pulmonary nodule localization with those of CT-guided Hook-wire localization. The aim is to explore the clinical application value of AI-assisted 3D reconstruction for pulmonary nodule localization in this surgical procedure.

## Materials and methods

### Patient data

This study conducted a retrospective analysis of 257 patients who underwent video-assisted thoracoscopic segmentectomy in the Department of Thoracic Surgery at Gansu Provincial People’s Hospital from January 2023 to August 2024. The inclusion criteria were as follows: (1) age > 18 years, with peripheral pulmonary nodules measuring ≤ 2 cm in diameter on CT and containing ground-glass components; (2) solitary or multiple nodules, but only a single surgical treatment was performed on the primary lesion; (3) absence of distant metastasis; (4) no prior neoadjuvant radiotherapy and complete clinical data. The exclusion criteria included: (1) poor cardiopulmonary function precluding tolerance of surgery; (2) presence of distant metastasis; (3) surgical procedures involving lobectomy or wedge resection.

In this study, surgeons selected one of the two localization methods based on the location of the patients’ pulmonary nodules, while also taking into account factors such as the patients’ own preferences and the opinions of their family members. All patients included in the study were clearly informed of the respective advantages and disadvantages of these two localization methods. Patients who chose AI-assisted three-dimensional (3D) reconstruction for pulmonary nodule localization were assigned to the AI-3D group, and those who underwent Hook-wire localization of pulmonary nodules were assigned to the Hook-wire group.

This study was approved by the Ethics Committee of Gansu Provincial People’s Hospital. All patients signed informed consent forms before surgery and confirmed their participation in this study.

### AI system description

**Figure 1:**
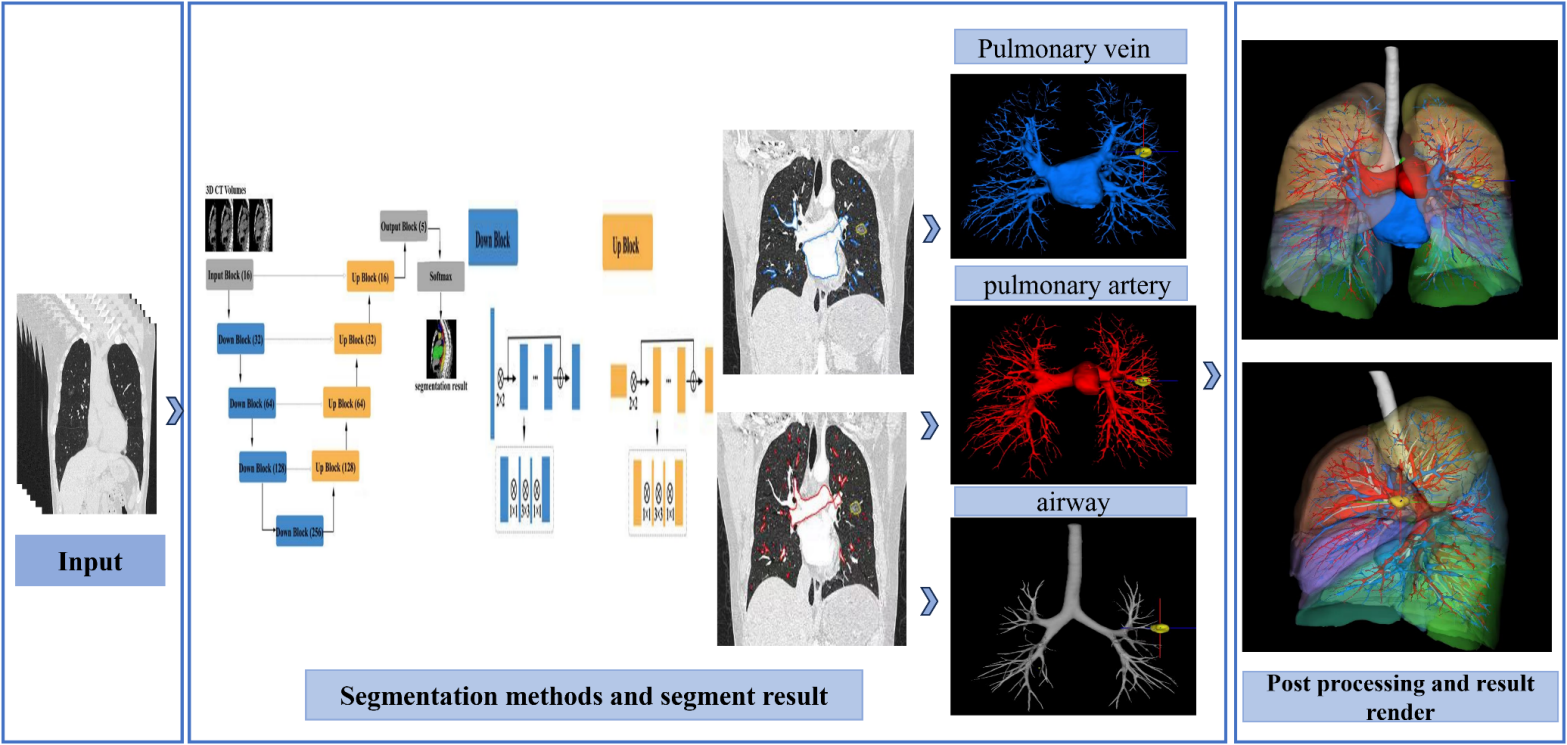
Schematic diagram of multi-resolution VB-Net architecture.

The chest CT 3D reconstruction software (uAI Surgical Planning Portal-Thoracic) used in this study is based on a multi-resolution VB-Nets architecture (**Figure 1**). This architecture achieves precise organ segmentation in medical images through a fully automated AI algorithm. It integrates a multi-resolution strategy, optimized loss function selection, and refined preprocessing and post-processing procedures. The multi-resolution VB-Nets architecture innovatively introduces a V-Net-based bottleneck structure, consisting of three convolutional layers. It effectively reduces the number of feature map channels through a 1×1×1 convolutional kernel, combines spatial convolution, and then restores the number of channels using another 1×1×1 convolutional kernel, significantly reducing the model size (e.g., from 250 MB to 8.8 MB) and accelerating the inference process.

To address the challenges of large 3D medical image volumes and high GPU memory consumption, the AI system in this study employs a multi-resolution strategy. Two VB-Net networks are trained separately: one focuses on low-resolution localization of the volume of interest (VOI), and the other focuses on high-resolution precise segmentation of organ boundaries. The generalized Dice loss function is used, which has the advantage of focusing on foreground voxels without being affected by the number of background voxels, making it suitable for both single-class and multi-class segmentation tasks.

**Figure 2:**
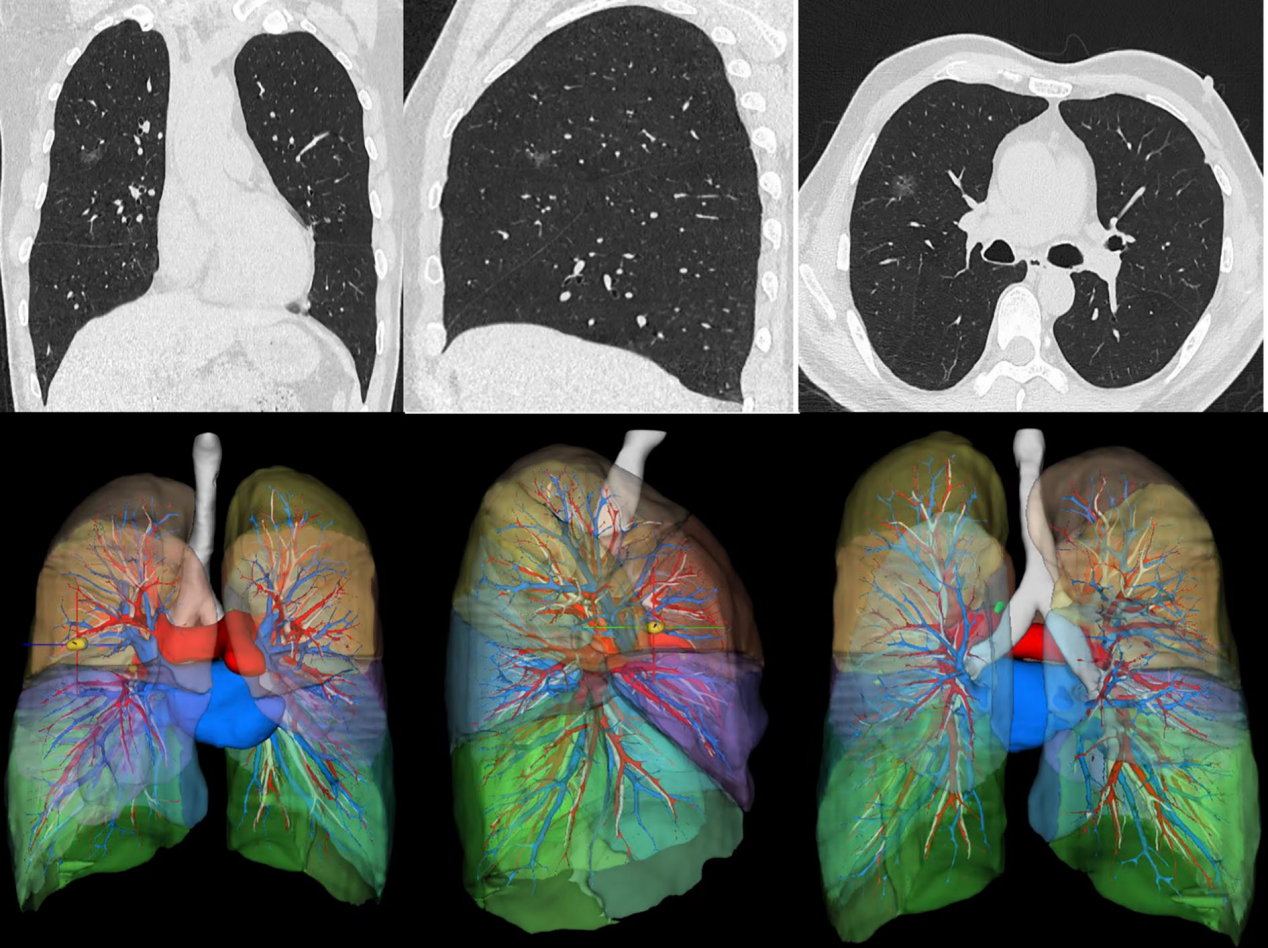
Schematic diagram of 3D reconstruction of chest CT using artificial intelligence.

During network training and inference, low-resolution and high-resolution segmentation networks are trained separately in this study, and they are fused using a multi-resolution strategy during the inference phase. First, the low-resolution network is used to localize the VOI, and then the high-resolution network is employed for precise segmentation. A fully convolutional network inference method is adopted to avoid the need for overlapping image patches, which is made possible by the multi-resolution strategy and a specially optimized inference engine. Finally, the largest 3D connected component is selected to remove isolated noise segments, and specific post-processing steps are taken for esophageal and cardiac segmentation to further enhance the accuracy and robustness of the segmentation. The 3D reconstruction results are shown in **Figure 2**.

### CT-guided hook-wire localization method for pulmonary nodules

**Figure 3:**
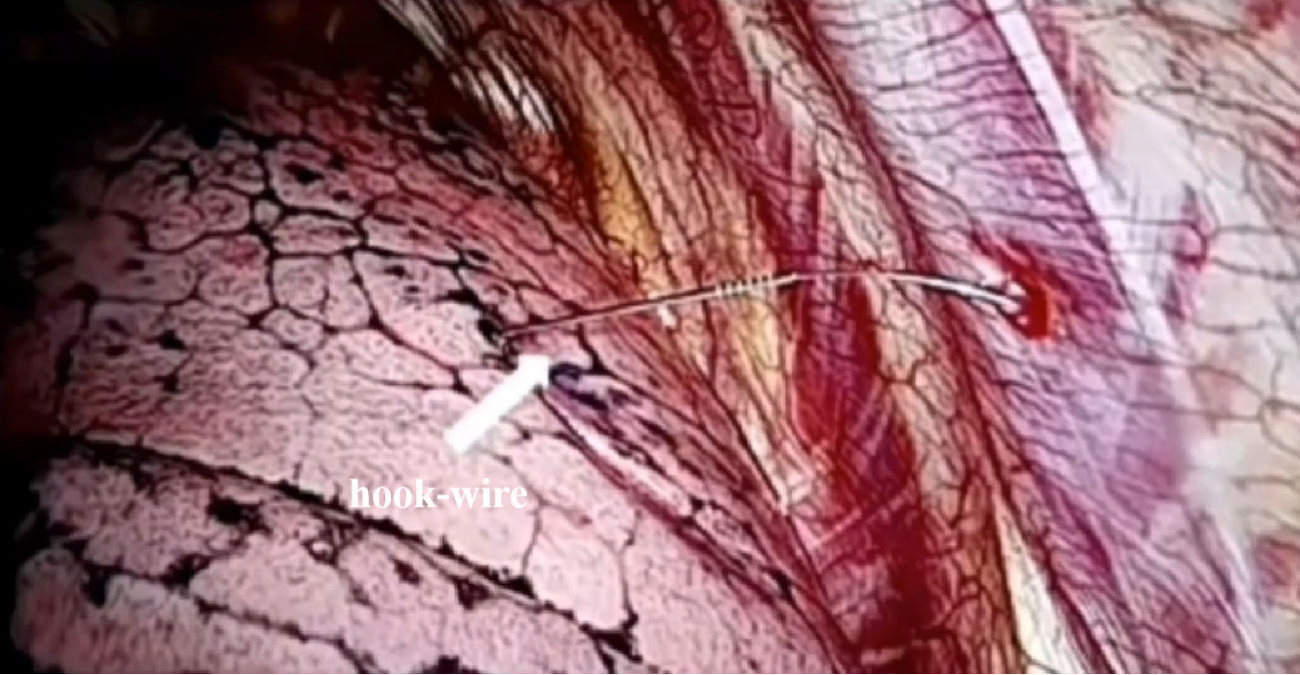
Localization of pulmonary nodules using a Hookwire needle.

The puncture plan was carried out 2 hours prior to surgery, with the radiologist performing the procedure based on the patient’s chest CT images and the surgical plan. First, the patient was positioned appropriately, and a routine CT plain scan was conducted to precisely determine the location of the target lesion and its relationship with adjacent tissues. Subsequently, the optimal needle insertion path was selected, and the needle insertion depth, the distance between the lesion and the chest wall, and the needle insertion angle were measured. The body surface entry point was then marked. After that, the surgical site was disinfected and draped. Local infiltration anesthesia was administered using 5 mL of 2% lidocaine. The puncture needle was inserted along the planned path into the extraparietal pleural space. Another CT scan was performed to confirm the direction of the puncture needle. The needle was then advanced to the lesion, and the Hook-wire localization wire was released. After withdrawing the outer sheath needle, a scan was conducted again to confirm that the wire was located within the pulmonary nodule, facilitating the intraoperative identification of the pulmonary nodule’s position (**Figure 3**).

### Surgical techniques

Under general anesthesia, ventilation of the healthy lung was achieved through double-lumen endotracheal intubation, and the patient was positioned in a lateral decubitus position on the healthy side. The surgery commenced with the creation of a small incision approximately 3 centimeters in length between the 5th and 6th intercostal spaces along the anterior axillary midline. During the procedure, intraoperative computer-assisted 3D reconstruction imaging technology was utilized in conjunction with thoracoscopy. An assistant, not at the operating table, rotated, displayed, or concealed the 3D reconstruction images in real-time according to the requirements of the chief surgeon, providing precise visual references. The segmentectomy adhered to the “unidirectional” surgical concept, progressing from the hilum in a unidirectional, layered manner from superficial to deep structures.

Throughout the surgery, 3D reconstruction images were employed to accurately locate the target segmental vessels and bronchi. The “inflation-deflation” method was used to precisely identify the intersegmental plane. A stapling device served as the primary tool for managing the intersegmental plane, ensuring precise and efficient cutting. During the segmentectomy, intra- and hilar lymph nodes were routinely cleared, and lobe-specific mediastinal lymph node dissection or systematic mediastinal lymph node sampling was performed as appropriate. To ensure the completeness and safety of the surgery, a margin of more than 2 centimeters or greater than the longest diameter of the tumor was maintained between the resection margin and the tumor. After resecting the pulmonary tissue, an incision was made to confirm the presence of the lesion, assess the adequacy of the resection margin, and obtain samples for intraoperative rapid frozen-section examination. Based on the frozen-section results, a decision was made on whether to proceed with further lobectomy. Routine hemostasis, water testing, and placement of a chest drainage tube were performed. After confirming the correct count of gauze pads and instruments, the chest wall incision was closed in layers, completing the surgery.

## Pulmonary function and quality of life assessment

### Pulmonary function assessment

Pulmonary function was evaluated using a spirometer (Power-Cube, Germany), measuring the percentage of forced vital capacity (FVC%) and the percentage of forced expiratory volume in one second (FEV1%). These tests were conducted one day before surgery and six months after surgery.

### Quality of life assessment

The patients’ quality of life was assessed using the Generic Quality of Life Inventory-74 (GQOLI-74). This questionnaire comprises 74 items divided into four dimensions and employs a 5-point scoring system, with higher scores indicating better quality of life. The assessment was also conducted one day before surgery and six months after surgery.

### Statistical analysis

Statistical analysis was performed using SPSS 26.0 software. Continuous variables were expressed as mean ± standard deviation ( $\bar{\text{X}}$±*s*), and comparisons between two independent samples were made using the independent samples t-test. Categorical variables were presented as frequencies and percentages (%), and intergroup comparisons were conducted using the chi-square test or Fisher’s exact test. A 1:1 PSM analysis was performed for the two groups of patients, with a caliper value set at 0.02. Matching factors included gender, age, body mass index (BMI), smoking history, nodule size, and nodule location. A *p*-value < 0.05 was considered statistically significant.

## Results

### PSM Results

Before PSM, there were significant differences in age and tumor location between the two groups of patients. PSM incorporated factors such as age, gender, body mass index (BMI), smoking history, nodule size, and nodule location. After 1:1 matching, there were 97 patients in the AI-3D group and 97 patients in the hook-wire group. No statistically significant differences were observed between the two groups for the six confounding factors. Detailed information is presented in **Table 1**. The specific distribution of the types of pulmonary segments to be resected is shown in **Table 2**.

**Table 1:** Comparison of baseline information between the two groups before and after PSM.

Characteristic	Before PSM		*P*	After PSM		P
	AI-3D group(134)	Hook-wire group(123)		AI-3D group(97)	Hook-wire group(97)	
Age (years)	57.37 ± 7.94	60.06 ± 8.43	*0.035*	57.78 ± 7.63	58.93 ± 8.24	*0.427*
Gender			*0.897*			*0.554*
Male	73 (54.48%)	68 (55.28%)		62 (63.92%)	58 (59.79%)	
Female	61 (45.52%)	55 (44.72%)		35 (36.08%)	39 (40.21%)	
BMI (kg/m2)	22.78 ± 2.11	22.36 ± 2.52	*0.672*	22.30 ± 2.34	22.58 ± 2.33	*0.575*
Smoking history			*0.763*			*0.530*
Yes	52 (38.81%)	50 (40.65%)		27 (27.84%)	31 (31.96%)	
No	82 (61.19%)	73 (59.35%)		70 (72.16%)	66 (68.04%)	
Nodule size (mm)	11.08 ± 3.13	10.89 ± 2.01	*0.591*	11.29 ± 3.27	10.96 ± 1.78	*0.449*
Distance from bronchus to nodule	24.91 ± 16.49	23.32 ± 15.76	*0.324*	24.45 ± 15.89	23.46 ± 16.01	*0.445*
Nodule location			*0.048*			*0.715*
Right upper lobe	44 (32.84%)	20 (16.26%)		27 (27.84%)	26 (26.80%)	
Right Middle lobe	11 (8.21%)	14 (11.38%)		8 (8.25%)	13 (13.40%)	
Right lower lobe	21 (15.67)	25 (20.33%)		21 (21.65%)	17 (17.53)	
Left upper lobe	36 (26.87%)	41 (33.33%)		25 (25.77%)	28 (28.87%)	
Left lower lobe	22 (16.42%)	23 (18.70%)		16 (16.49%)	13 (13.40%)	

**Table 2:** Lung subsegmental resection sites.

Type of planned segmentectomy	AI-3D group (n=97)	Hook-wire group (n=97)
Left lung	42 (43.30%)	45 (46.40%)
S1 + 2	9 (9.28%)	9 (9.28%)
S1 + 2c	5 (5.15%)	4 (4.12%)
S1 + 2+3	4 (4.12%)	6 (6.19%)
S3	3 (3.09%)	5 (5.15%)
S4 + 5	7 (7.22%)	8 (8.25%)
S6	6 (6.19%)	9 (9.28%)
S8	5 (5.15%)	4 (4.12%)
S8 + 9+10	2 (2.06%)	0
S9 + 10	1 (1.03%)	0
Right lung	55 (56.70%)	53 (54.60%)
S1	14 (14.43%)	12 (12.37%)
S2	11 (11.34%)	12 (12.37%)
S2 + 1a	5 (5.15%)	3 (3.09%)
S3	7 (7.22%)	4 (4.12%)
S6	8 (8.25%)	5 (5.15%)
S8	6 (6.19%)	8 (8.25%)
S9	3 (3.09%)	5 (5.15%)
S10	1 (1.03%)	4 (4.12%)

### Surgical outcomes

After PSM, the surgical data for the two patient groups are presented in **Table 3**. Both groups successfully completed the surgeries without conversion to thoracotomy, and there were no cases of perioperative mortality. In the Hook-wire group, 2 cases of pneumothorax and 4 cases of hemothorax occurred, while the AI-3D group had no complications of pneumothorax or hemothorax. Compared with the Hook-wire group, the AI-3D group had a shorter operative time (*P* = 0.002), fewer days of postoperative chest tube indwelling (*P* < 0.001), and a shorter total postoperative hospital stay (*P* = 0.026). Additionally, this group had less intraoperative blood loss (*P* < 0.001) and lower postoperative chest tube drainage volume (*P* = 0.003), with all differences being statistically significant. There were no significant differences between the two groups in the incidence of postoperative complications (*P* = 0.470) and the number of dissected lymph nodes (*P* = 0.357). Detailed information is shown in **Table 3**.

**Table 3:** Surgical data of patients [cases(%)/ $\bar{\text{x}}$± s].

Characteristic	AI-3D group(n=97)	Hook-wire group(n=97)	*P*
Pneumothorax	0	2 (2%)	
Hemothorax	0	4 (4%)	
Operative time (min)	132.19±24.02	153.16±28.9	*0.002*
Intraoperative bleeding (ml)	42.19±13.99	71.57±17.91	*P<0.001*
Conversion to thoracotomy	0	0	
Number of lymph node dissection	7.34±2.13	7.27±2.07	*0.357*
Total postoperative chest drainage	394.27±39.93	432.03±45.78	*0.003*
postoperative chest tube stay (day)	2.8±1.3	4.24±1.7	*P<0.001*
Postoperative hospital stay (day)	5.15±1.10	6.26±1.13	*0.026*
Postoperative complications			*0.470*
Yes	8 (8.25)	11 (11.34)	
No	89 (91.75)	86 (88.66)	

### Comparison of pulmonary function and quality of life

On the day before surgery, no significant differences were observed in the percentage of forced vital capacity (FVC%) and the percentage of forced expiratory volume in one second (FEV1%) between the two groups of patients. At six months postoperatively, the AI-3D group exhibited higher FVC% and FEV1% values compared to the control group (**Table 4**).

**Table 4:** Comparison of changes in lung function between the two groups ( $\bar{\text{x}}$±s).

Factors	Pulmonary function test time	AI-3D group (n=97)	Hook-wire group (n=97)	*P*
FVC%	One day before surgery	87.63±4.13	88.03±4.27	*0.339*
	Six months after surgery	80.28±3.26	77.43±3.77	*0.002*
FEV1%	One day before surgery	89.33±4.37	90.04±4.23	*0.458*
	Six months after surgery	83.67±3.45	78.19±4.13	*<0.001*

Furthermore, on the day before surgery, there were no significant differences in the scores of psychological function, physical function, social function, and material living conditions between the two groups. At six months postoperatively, the AI-3D group demonstrated significant advantages in the scores of psychological function, physical function, social function, and material living conditions (**Table 5**).

**Table 5:** Comparison of preoperative and postoperative quality of life between the two groups ( $\bar{\text{x}}$±s).

Factors	Time	AI-3D group	Hook-wire group	*P*
Psychological function	One day before surgery	59.30±4.26	60.02±4.53	*0.358*
	Six months after surgery	81.05±3.42	76.57±3.66	*<0.001*
Somatic function	One day before surgery	60.73±3.89	61.13±4.35	*0.306*
	Six months after surgery	84.51±8.01	81.27±7.43	*0.002*
Social function	One day before surgery	63.13±3.27	62.78±3.41	*0.459*
	Six months after surgery	84.15±6.38	80.18±4.56	*<0.001*
Material state	One day before surgery	60.78±2.24	60.34±3.78	*0.263*
	Six months after surgery	85.27±6.36	82.33±6.46	*0.003*

## Discussion

Segmentectomy is a safe therapeutic option for early-stage lung cancer (limited to T1bN0). Compared with traditional lobectomy, segmentectomy can achieve similar therapeutic effects while preserving more pulmonary function and improving patients’ postoperative quality of life (2, 12). Segmentectomy requires the identification of tertiary and quaternary bronchi along with their accompanying arteries and veins; however, there is significant anatomical variation in the vascular structures at the segmental level (13, 14). During the procedure, it is crucial to avoid damaging major blood vessels and bronchi to prevent severe postoperative complications such as hemorrhage, atelectasis, and bronchopleural fistula. Accurate and rapid identification of pulmonary nodules is therefore essential for the safe and effective implementation of anatomical segmentectomy. This study aimed to compare the clinical benefits of artificial intelligence-assisted 3D reconstruction of pulmonary nodules with CT-guided hook-wire localization in segmentectomy, with a particular focus on patients’ postoperative pulmonary function and quality of life recovery.

Early-stage lung cancer lesions are typically small, and intraoperative localization of some subsolid nodules can be extremely challenging. Relying solely on palpation or instrument sliding for localization has a success rate of only 30% (15–17). Therefore, preoperative adjuvant lesion localization is of paramount importance. Currently, commonly used preoperative localization methods in clinical practice include percutaneous hook-wire localization (18), electromagnetic navigation bronchoscopy-guided puncture localization (19), and CT-based 3D reconstruction-assisted localization. Each of these methods has its own advantages and disadvantages. Percutaneous hook-wire localization is simple, quick, and accurate but can be difficult to apply for pulmonary nodules located in special areas such as the apex of the lung or beneath the scapula due to obstruction by important blood vessels, nerves, or the scapula within the chest wall. Additionally, it carries risks of complications such as pneumothorax and hemothorax, necessitating immediate surgery after localization. Electromagnetic navigation bronchoscopy-guided puncture localization can reach anatomically inaccessible regions and is associated with fewer complications; however, its clinical application is limited by cost-effectiveness considerations and a lack of standardization in practice. In this context, CT-based 3D reconstruction-assisted localization has emerged as a method that balances accuracy and safety.

In recent years, three-dimensional (3D) visualization and reconstruction technologies have gradually been integrated into the daily practice of thoracic surgery. Compared with traditional imaging modalities such as X-rays and CT scans, they offer significant advantages in terms of intuitiveness, 3D stereopsis, and precision (20). Previous semi-automatic 3D reconstruction tools (e.g., Mimics^®^ software), which were based on the stacking of two-dimensional (2D) images from contrast-enhanced CT scans and employed threshold segmentation methods, required substantial time and effort to construct 3D models. The complex and time-consuming software operations somewhat diminished surgeons’ enthusiasm for conducting 3D reconstructions (12–14).

This study is based on an AI system powered by deep learning. The system employs fully automated algorithms to achieve 3D reconstruction from contrast-enhanced CT scan images. It is highly modular, with each module dedicated to segmenting specific pulmonary tissues and capable of independent operation and optimization. By integrating deep learning, data-driven approaches, and a clinical feedback error learning mechanism, the system requires minimal manual intervention to function. Our team’s previous research has confirmed the accuracy and safety of CT-assisted 3D visualization technology in pulmonary nodule resection (21). We chose to observe the recovery of pulmonary function at the sixth month postoperatively (22). The results revealed that both the forced vital capacity percentage (FVC%) and the forced expiratory volume in one second percentage (FEV1%) at six months postoperatively were higher in the 3D reconstruction group than in the Hook-wire group, indicating that patients in the AI-3D group experienced less short-term impairment in pulmonary function compared to those in the Hook-wire group. Studies have shown that postoperative pulmonary function recovery is closely related to residual lung compensation, and postoperative pulmonary function exercises can promote the expansion and compensation of the residual lung, facilitating short-term postoperative pulmonary function recovery (23, 24).

To control the impact of anatomical complexity of cases and variations in surgeons' experience on the study results, this study adopted dual measures: ① We conducted quantitative screening to include peripheral pulmonary nodules with a diameter of less than 2 cm in the study group, ensuring baseline comparability of tumor anatomical characteristics between the two groups; ② All surgical procedures were performed by the same highly experienced surgeon (with 15 years of experience in thoracoscopic surgery and completing ≥100 segmentectomy procedures annually), with consistent surgical team members to minimize variations in surgical skills and team coordination fluctuations. We chose to observe the recovery of pulmonary function at the sixth month postoperatively (25). The results revealed that both the forced vital capacity percentage (FVC%) and the forced expiratory volume in one second percentage (FEV1%) at six months postoperatively were higher in the 3D reconstruction group than in the Hook-wire group, indicating that patients in the AI-3D group experienced less short-term impairment in pulmonary function compared to those in the Hook-wire group. Studies have shown that postoperative pulmonary function recovery is closely related to residual lung compensation, and postoperative pulmonary function exercises can promote the expansion and compensation of the residual lung, facilitating short-term postoperative pulmonary function recovery (26, 27).

However, the compensatory capacity of the residual lung is limited, and postoperative pulmonary function recovery gradually stabilizes and reaches saturation over time. Therefore, the long-term impact of 3D reconstruction technology on patients’ pulmonary function still requires further validation through long-term follow-up observations. Additionally, our assessment of the scores on the Generic Quality of Life Inventory-74 (GQOLI-74) at six months postoperatively showed that the AI-3D group scored higher across all dimensions, indicating that preoperative enhanced chest CT 3D reconstruction contributes to the recovery of patients’ postoperative quality of life.

This study has the following limitations: (1) Although artificial intelligence has achieved stability through deep learning of vast clinical datasets, imaging quality remains a crucial factor affecting reconstruction accuracy. To mitigate the impact of CT artifacts, the system enhances its artifact resistance by integrating multi-resolution VB-Net with an attention mechanism. Additionally, it establishes standardized acquisition protocols and converts multi-vendor data into a uniform format via DICOM standards to minimize device-related variations.(2) Our dataset is derived from East Asian populations. Future studies should incorporate more diverse ethnic groups to further validate the model’s generalization capability.(3) Retrospective studies are limited by recall bias and sample size constraints. Prospective multicenter randomized controlled trials should be conducted in the future to further validate our conclusions. These trials should include long-term follow-up to assess long-term pulmonary function and quality of life, incorporating hard endpoints such as 5-year disease-free survival and local recurrence rates to ensure a comprehensive evaluation of treatment efficacy.

In summary, the application of artificial intelligence-assisted chest CT 3D reconstruction technology in segmentectomy enables precise localization of lesions and preoperative visualization of the anatomical structure of pulmonary segments. It helps shorten operative time, reduce intraoperative blood loss, avoid excessive dissection, mitigate the impact on postoperative pulmonary function, and promote good recovery of postoperative quality of life. Additionally, this technology avoids the trauma associated with needle localization. In the future development of digital, precise, and personalized medicine, this technology holds broad application prospects.

## Data Availability

The original contributions presented in the study are included in the article/Supplementary Material. Further inquiries can be directed to the corresponding author/s.
